# Protective Role of AMP-Activated Protein Kinase-Evoked Autophagy on an In Vitro Model of Ischemia/Reperfusion-Induced Renal Tubular Cell Injury

**DOI:** 10.1371/journal.pone.0079814

**Published:** 2013-11-06

**Authors:** Li-Ting Wang, Bo-Lin Chen, Cheng-Tien Wu, Kuo-How Huang, Chih-Kang Chiang, Shing Hwa Liu

**Affiliations:** 1 Institute of Toxicology, College of Medicine, National Taiwan University, Taipei, Taiwan; 2 Department of Urology, College of Medicine, National Taiwan University, Taipei, Taiwan; 3 Departments of Integrated Diagnostics & Therapeutics and Internal Medicine, National Taiwan University Hospital and National Taiwan University College of Medicine, Taipei, Taiwan; 4 Department of Pediatrics, National Taiwan University Hospital, Taipei, Taiwan; 5 Department of Medical Research, China Medical University Hospital, China Medical University, Taichung, Taiwan; Aarhus University, Denmark

## Abstract

Ischemia/reperfusion (I/R) injury is a common cause of injury to target organs such as brain, heart, and kidneys. Renal injury from I/R, which may occur in renal transplantation, surgery, trauma, or sepsis, is known to be an important cause of acute kidney injury. The detailed molecular mechanism of renal I/R injury is still not fully clear. Here, we investigate the role of AMP-activated protein kinase (AMPK)-evoked autophagy in the renal proximal tubular cell death in an *in vitro* I/R injury model. To mimic *in vivo* renal I/R injury, LLC-PK1 cells, a renal tubular cell line derived from pig kidney, were treated with antimycin A and 2-deoxyglucose to mimic ischemia injury followed by reperfusion with growth medium. This I/R injury model markedly induced apoptosis and autophagy in LLC-PK1 cells in a time-dependent manner. Autophagy inhibitor 3-methyladenine (3MA) significantly enhanced I/R injury-induced apoptosis. I/R could also up-regulate the phosphorylation of AMPK and down-regulate the phosphorylation of mammalian target of rapamycin (mTOR). Cells transfected with small hairpin RNA (shRNA) for AMPK significantly increased the phosphorylation of mTOR as well as decreased the induction of autophagy followed by enhancing cell apoptosis during I/R. Moreover, the mTOR inhibitor RAD001 significantly enhanced autophagy and attenuated cell apoptosis during I/R. Taken together, these findings suggest that autophagy induction protects renal tubular cell injury via an AMPK-regulated mTOR pathway in an *in vitro* I/R injury model. AMPK-evoked autophagy may be as a potential target for therapeutic intervention in I/R renal injury.

## Introduction

Ischemia/reperfusion (I/R) injury is a common cause of injury to target organs and contributes to several important diseases, such as myocardial infarction, hypovolemic shock, thromboembolism, and acute kidney injury (AKI) [1-4]. Ischemic injury is caused by an initial shortage of blood supply, while the injury associated with reperfusion develops over hours to days after the initial insult. In the kidneys, I/R injury is known to be an important cause of AKI. It occurs in several clinical conditions such as renal transplantation, trauma, and sepsis [5]. Renal I/R has been demonstrated to cause variant pathological changes [6-8] including tubular injury that leads to the induction of inflammatory responses [9,10], increase of vasoconstriction [11,12], and decrease of vasodilation [13]. The detailed molecular mechanisms of renal I/R injury are still not fully clear.

AMPK, a heterotrimeric complex, consisting of a catalytic α-subunit and regulatory β- and γ-subunits with three isoforms, is abundantly expressed in the kidneys [14]. AMPK is also known to be involved in renal pathophysiology including podocyte function modulation [15], diabetes-induced renal hypertrophy [16], and polycystic kidney disease [17]. Oxidative stress and aging have also been suggested to influence AMPK expression in kidney [18]. The activation of AMPK negatively regulated metabolism, cell growth, proliferation or autophagy [19,20]. Moreover, AMPK activation down-regulates the signaling of mammalian target of rapamycin (mTOR) [21], which is a major positive stimulus for cellular stress-regulated protein synthesis, cell growth, and cell size. The mTOR signaling pathway is also known to negatively regulate the autophagy [22]. The AMPK-regulated mTOR signaling pathway was considered an important regulator of autophagy during energy depletion [23,24]. AMPK has been demonstrated to improve the ventricular function after cardiac I/R injury [25]. Evidence has also shown that autophagy participates in the renal I/R injury [26]. However, the roles of AMPK signaling and autophagy induction in the renal I/R injury are still not fully understood and need to be clarified. In this study, we aimed to clarify the potential role of AMPK-regulated mTOR signaling pathway in autophagy induction and renal tubular cell injury during *in vitro* I/R. To mimic the *in vivo* renal I/R injury, a renal proximal tubular cell line LLC-PK1 derived from pig kidney were treated with a mitochondrial respiration inhibitor (antimycin A) and a non-metabolizable glucose analog (2-deoxyglucose) to induce ischemia injury followed by reperfusion with growth medium [27,28]. The results suggest that autophagy protects renal tubular cell injury via an AMPK-regulated mTOR pathway in an *in vitro* I/R injury model.

## Materials and Methods

### Materials

 Antimycin A, 2-deoxy-D-glucose (2-deoxyglucose), RAD001 (mTOR inhibitor), and 3-methyladenine (3MA; autophagy specific inhibitor) were purchased from Sigma-Aldrich (St. Louis, MO, USA). Rapamycin was purchased from Calbiochem (Bad Soden, Germany). Compound C (AMPK inhibitor) was purchased from Merck (Darmstadt, Germany).

### Cell Culture

LLC-PK1 cells, an established renal proximal tubular cell line derived from pig kidney, were purchased from American Type Culture Collection (ATCC) and cultured in growth medium consisting of medium 199 (M199; GIBCO, Grand Island, NY, USA) supplemented with 3% fetal bovine serum (FBS) and 1% antibiotics (100 IU/ml penicillin, 100 µg/ml streptomycin) at 37°C under 5% CO_2_. NRK-52E cells were purchased from the Bioresource Collection and Research Center (Hsinchu, Taiwan). NRK-52E cells were cultured in DMEM (GIBCO, Grand Island, NY, USA) supplemented with 5% FBS and 1% antibiotics (100 IU/ml penicillin, 100 µg/ml streptomycin) at 37°C under 5% CO_2_.

### *In vitro* I/R injury model

LLC-PK1 cells were incubated in a Krebs-Henseleit (KH) buffer (115 mM NaCl, 3.6 mM KCl, 1.3 mM KH_2_PO_4_, 25 mM NaHCO_3_, 1 mM CaCl_2_, 1 mM MgCl_2_, pH =7.4) with antimycin A (a complex III inhibitor of mitochondrial electron transport; 12.5 to 100 μM) and 2-deoxyglucose (a nonmetabolizable isomer of L-glucose and a glycolysis inhibitor; 5 mM) for 1.5 h to induce *in vitro* ischemia injury [27,28]. In some experiments, NRK-52E, a normal rat renal tubular cell line, cells were incubated in a Krebs-Henseleit (KH) buffer with antimycin A (5 μM) and 2-deoxyglucose (5 mM) for 1 h to induce *in vitro* ischemia injury. The reperfusion was achieved in LLC-PK1 cells and NRK-52E cells by washing with KH buffer and then cultured in complete growth medium for various time courses. 

### Sub-G1 analysis for fragmented DNA

LLC-PK1 cells were exposed to vehicle or antimycin A plus 2-deoxyglucose for 1.5 h and then followed by reperfusion for 24 h. Subsequently, the cells were suspended into PBS and incubated with 0.1 mg/ml of RNaseA (Invitrogen, Carlsbad, CA, USA) and 10 μg/ml of PI (Sigma-Aldrich) for 20 min. Flow cytometric analysis was performed using Becton Dickinson FACSCalibur cytometer with an argon ion laser (488 nm) as the excitation light. Cell Quest version 6.0 software was used for DNA content analysis.

### PI and Annexin V assays for apoptosis detection

After *in vitro* I/R treatment, LLC-PK1 cells were collected in different reperfusion time points and then washed with PBS twice. Following centrifugation, the PBS was discarded and the cells were stained with Annexin V-FITC and PI staining kit (BD Biosciences) for 15 min at room temperature in the dark as previously described [29]. Flow cytometric analysis was performed using Becton Dickinson FACSCalibur cytometer. Both early (Annexin V-positive, PI-negative) apoptotic cells and late (Annexin V-positive and PI-positive) apoptotic cells were analyzed. Totally, 10,000 cells were analyzed per sample.

### Analysis of autophagy by green fluorescent protein (GFP)-cytosolic microtubule-associated protein light chain 3 (LC3) distribution and monodansylcadaverine (MDC) staining

The transient transfection in LLC-PK1 cells was performed by the Lipofectamine 2000 reagent (Invitrogen) according to the manufacture's recommendations. The cells were transfected with a control pcDNA6.2 vehicle or a GFP-LC3 fusion protein expression vector (pcDNA6.2-Em GFP-LC3). After *in vitro* I/R treatment, the transfected cells were harvested and stained with Hoechst33258 (1 μg/ml; Sigma-Aldrich). For MDC staining, the cells were treated with 50 μM MDC (Sigma-Aldrich) in the medium and incubated at 37°C for 20 min. The localizations of LC3 and autophagosome formations were examined by fluorescence microscopy.

### Western blotting analysis

Western blotting analysis was performed as previously described [30]. After treatment, cells were collected and lysed by RIPA buffer (Santa Cruz Biotechnology, Santa Cruz, CA, USA) and then centrifuged at 10,000×g for 20 min at 4°C. The supernatant solution was determined by bicinchoninic acid (BCA) protein assay reagent (Thermo Fisher Scientific, Dreieich, Germany). Equal amounts of proteins (40 µg) were separated by 6-15% SDS–polyacrylamide gel electrophoresis. The proteins were electrophoretically transferred to a polyvinylidene difluoride membrane and blocked with 5% fat-free milk in Tris-buffered saline/Tween-20 (TBST) buffer (20 mM Tris, 150 mM NaCl, 0.01% Tween-20, pH 7.5) for 1 h. The primary antibodies for caspase-3 (Cell Signaling Technology, Beverly, MA, USA), phospho-AMPKα (Cell Signaling Technology), AMPKα1 (Abcam), AMPKα2 (Abcam), phospho-mTOR (Cell Signaling Technology), LC3 (Cell Signaling Technology), Bax (GeneTex Inc., Irvine, CA, USA) and β-actin (Santa Cruz) were incubated overnight at 4°C. After washing for three times, membranes were reacted with secondary goat anti-rabbit or anti-mouse horseradish peroxidase (HRP)-conjugated antibodies. The signals were visualized by an enhanced chemiluminescence reagents (Millipore Corporation, Billerica, MA, USA) detection system and recorded on X-film. Finally, densitometric analysis was performed using Scion Image software (Scion, Frederick, MD, USA) to quantify protein expression, and the results were normalized to the control group [31,32]. 

### Lentivirus infection of short hairpin RNA (shRNA) for AMPK

The shRNAs were purchased from Open Biosystems, Thermo (Taipei, Taiwan). The LLC-PK1 cells were infected with lentivirus expressing the targeting shRNA for AMPKα1 or scrambled as per the manufacturer’s instructions. The target sequences of AMPKα1 shRNA is presented as 5’-CTTGAAATGTGTGCAAATCTAA-3’. Cells were treated with hexadimethrine bromide (10 μg/ml) and lentiviral particles (40 μl) for 24 h, and then replaced with fresh medium. The stable expressing cells were selected using 2 μl/ml puromycin.

### Statistical analysis

Data are presented as means ± SDs. The significant difference from the respective controls for each experimental test condition was assessed by one-way analysis of variance (ANOVA) and Student's t-test. The difference is significant if the *P*-value is less than 0.05 or 0.01. 

## Results

### Apoptosis was induced in an *in vitro* I/R injury model

**Figure 1:**
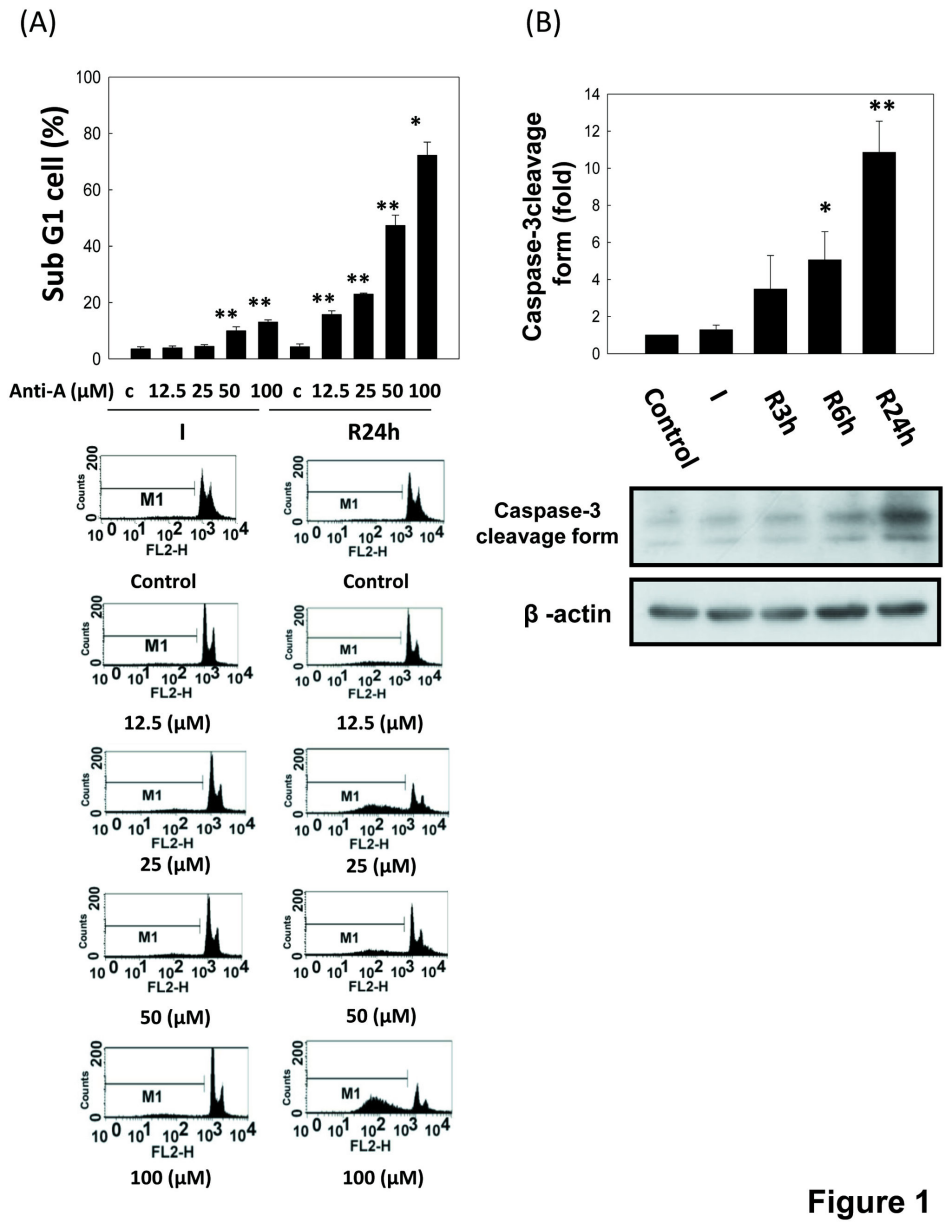
Cell injury and caspase 3 cleavage in renal tubular LLC-PK1 cells during *in* *vitro* I/R. Cells were treated with antimycin A (12.5 to 100 μM) and 5 mM 2-deoxyglucose for 1.5 h to induce ischemia (I) injury followed by reperfusion (R) with growth medium for 24 h. Percentages of cells with the hypodiploid DNA content (sub-G1 cells) were determined by flow cytometry (A). Moreover, the protein expression of caspase-3 cleavage form was determined by Western blotting (B). Data are presented as the means ± SDs in three independent experiments. *P < 0.05 and **P < 0.01 as compared with vehicle control group. C: control. Anti-A: Antimycin A.

**Figure 2:**
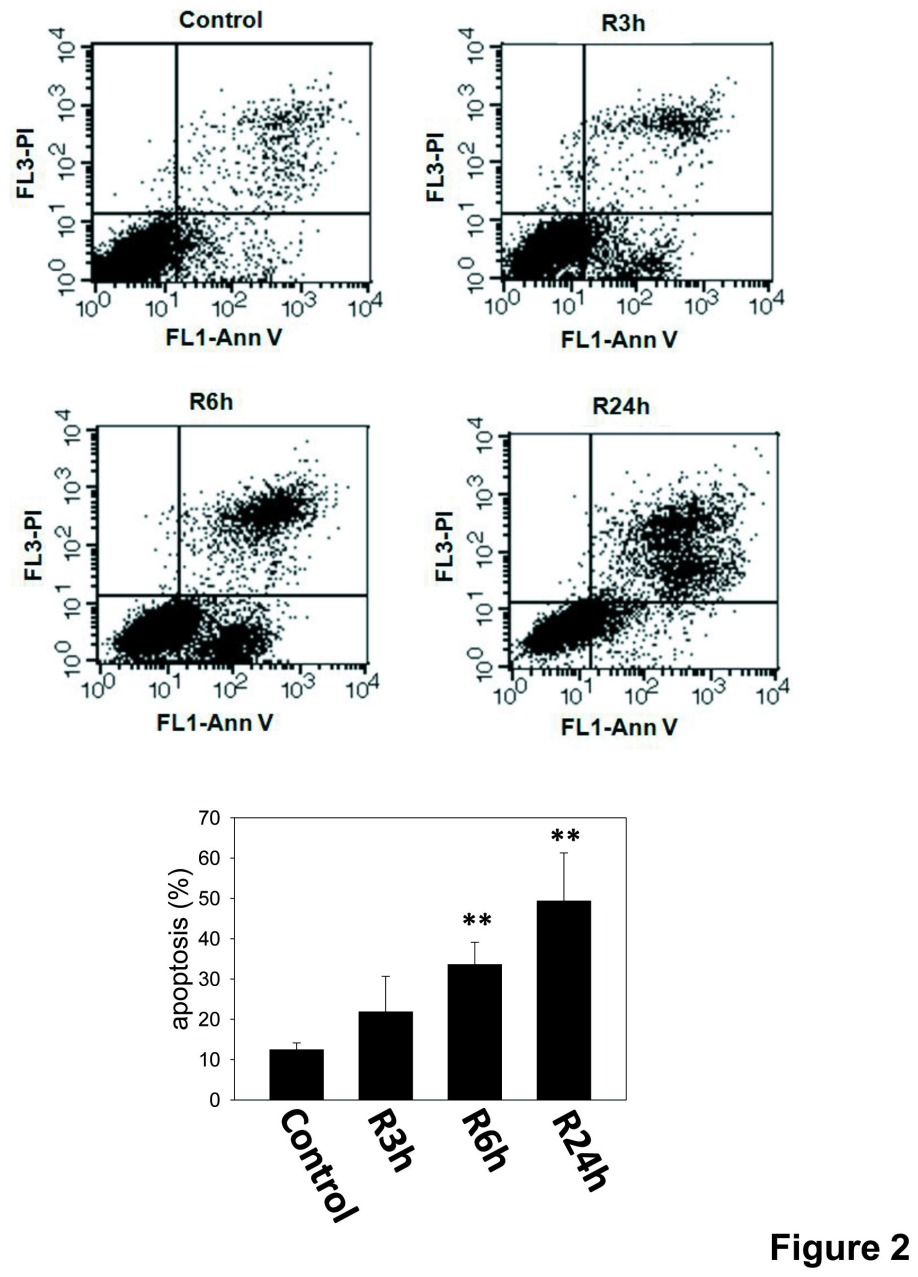
Apoptosis in renal tubular LLC-PK1 cells during *in* *vitro* I/R. Cells were treated with antimycin A (12.5 to 100 μM) and 5 mM 2-deoxyglucose for 1.5 h to induce ischemia (I) injury followed by reperfusion (R) with growth medium for 24 h. Cell apoptosis was performed by Annexin V and PI dual staining and determined by flow cytometry. Data are presented as the means ± SDs in three independent experiments. **P < 0.01 as compared with vehicle control group. Ann V: annexin v.

 To evaluate the effects of I/R on renal cell apoptosis, LLC-PK1 cells were treated with antimycin A (12.5 to 100 μM) and 5 mM 2-deoxyglucose to mimic the *in vivo* I/R reaction [27,28]. As shown in Figure 1A, the sub-G1 cell percentages at reperfusion 24 h after ischemia period were obviously higher than ischemia period in an antimycin A dose-dependent manner. When cells were treated with 50 μM antimycin A and 5 mM 2-deoxyglucose for inducing ischemia, cells significantly caused approximately 50% sub-G1 cells at reperfusion 24 h. The increase of caspase-3 cleavage was also shown in LLC-PK1 cells during I/R (Figure 1B). Furthermore, the annexin V/PI staining showed that the apoptotic cells are markedly increased during I/R (Figure 2). Results indicated that I/R injury induced LLC-PK1 cell apoptosis in a time-dependent manner. 

### Autophagy was induced in an *in vitro* I/R injury model

**Figure 3:**
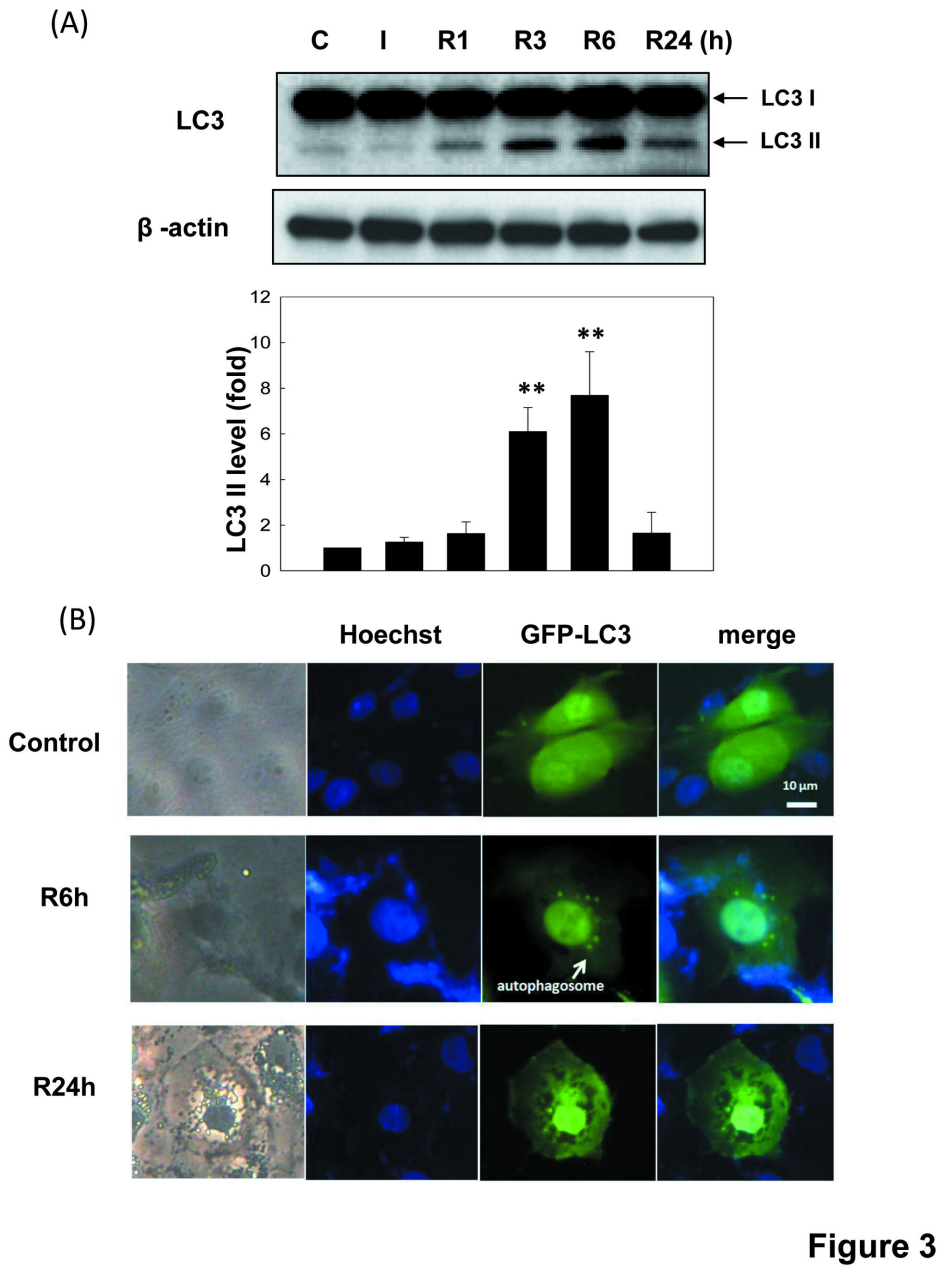
Autophagy in LLC-PK1 cells during *in* *vitro* I/R. Cells were treated with 50 μM antimycin A and 5 mM 2-deoxyglucose for 1.5 h to induce ischemia (I) injury followed by reperfusion (R) for 1-24 h. In figure 3A, autophagy was determined by Western blotting using anti-LC3 antibody. The β-actin was used to an internal control. Data are presented as the means ± SDs in three independent experiments. *P < 0.05 and **P < 0.01 as compared with vehicle control group. In figure 3B, the GFP-LC3 puncta formation in LLC-PK1 cells was determined by immunofluorescence. Cells were transiently transfected with GFP-LC3 for 4 h before I/R treatment. Arrow indicates GFP-LC3 puncta formation (green). Nuclei were stained by Hoechst33258 dye (blue). Scale bar = 10 μm. Results shown are representative of at least three independent experiments.

**Figure 4:**
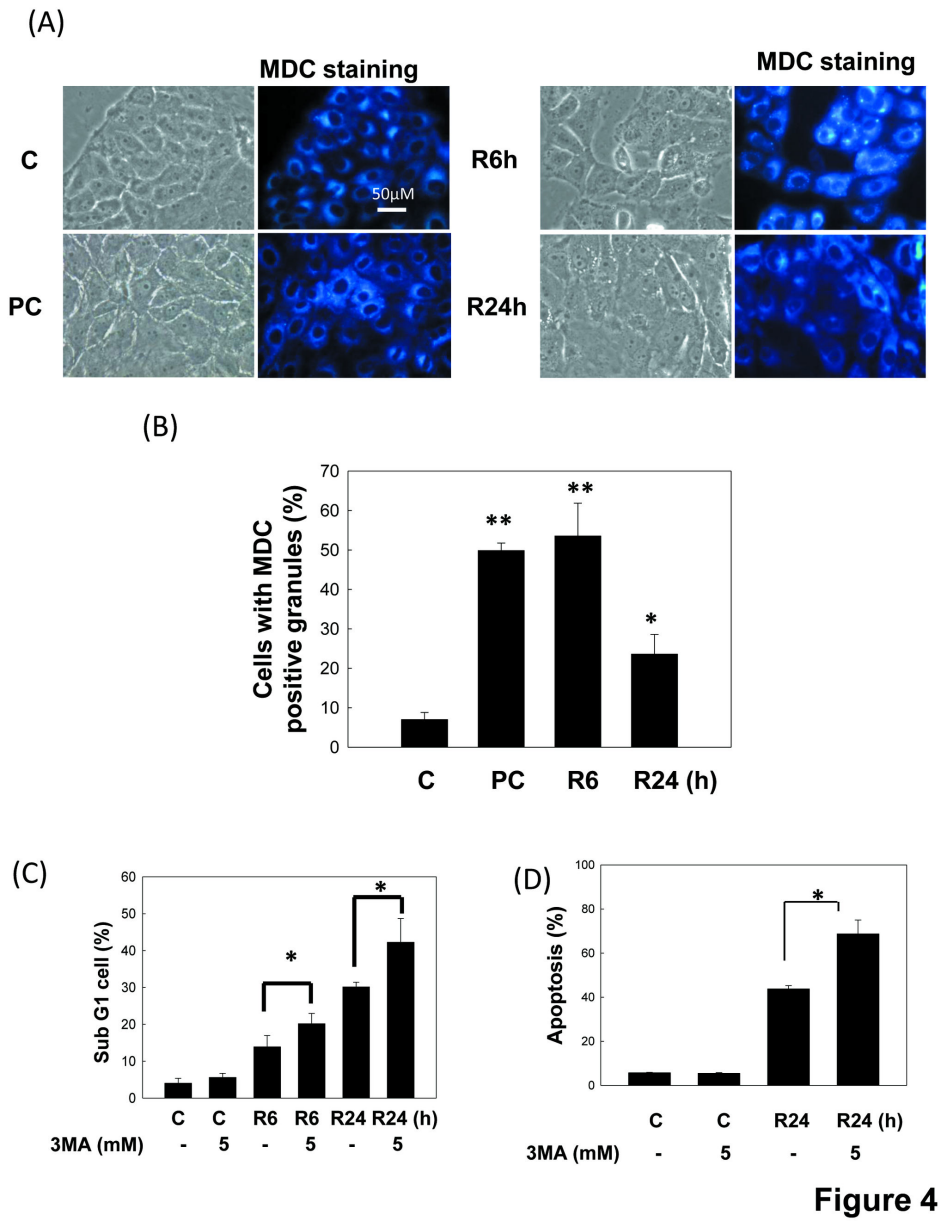
Enhancement of apoptosis by autophagy inhibitor in LLC-PK1 cells during *in* *vitro* I/R. Cells were treated with 50 μM antimycin A and 5 mM 2-deoxyglucose for 1.5 h to induce ischemia (I) injury followed by reperfusion (R) for 6 or 24 h in the absence or presence of 5 mM 3MA. Cells were treated with 1 μM rapamycin for 6 h as an autophagic positive control. The MDC staining for autophagic vacuoles was examined by fluorescence microscopy (A). The number of MDC-positive cells was counted (a minimum of 100 cells per sample) (B). Moreover, the percentages of cells with the hypodiploid DNA content (sub-G1 cells) were determined by flow cytometry (C). Cell apoptosis was also performed by Annexin V and PI dual staining and determined by flow cytometry (D). In B, C and D, data are presented as the means ± SDs in three independent experiments. *P < 0.05 and **P < 0.01 as compared with vehicle control group. PC: positive control. C: control.

To investigate whether autophagy takes a part in I/R-induced renal cell injury, we examined autophagy induction in LLC-PK1 cells in an *in vitro* I/R injury model. LC3 activation is commonly used to monitor autophagy and the mount of LC3-II is clearly correlated with the number of autophagosomes [33]. We observed this feature to detect autophagy induction. As shown in Figure 3A, I/R activated the LC3-II formation in LLC-PK1 cells in a time-dependent manner. The LC3-II formation was not obvious during the ischemia period, but was significantly enhanced during reperfusion period. Moreover, GFP-labeled LC3-transfected cells were used to examine the LC3 localization. Results showed that GFP-LC3 green dots are obviously distributed in cytoplasm (Figure 3B). The autophagosome formation was further observed using MDC staining [34]. MDC positive cells were markedly increased in LLC-PK1 cells during I/R (Figures 4A and 4B). Cells were treated with 1 μM rapamycin for 6 h as a positive control. Results indicated that I/R could evoke autophagy in the renal proximal renal cells.

We next investigated the role of autophagy in an *in vitro* I/R injury model. A pharmacological inhibitor of autophagy 3MA [35] was used to determine the effect of autophagy on cell apoptosis. As shown in Figure 4C, when LLC-PK1 cells were pre-treated with 5 mM 3MA, the percentages of sub-G1 cells were significantly elevated as compared with non-treated cells at reperfusion 24 h (Figure 4C). The annexin V/PI staining showed similar results as the Sub-G1 analysis (Figure 4D). Results showed that autophagy induction might protect renal cells from apoptosis in an *in vitro* I/R injury model.

### Changes in the phosphorylations of AMPK and mTOR in an *in vitro* I/R injury model

**Figure 5:**
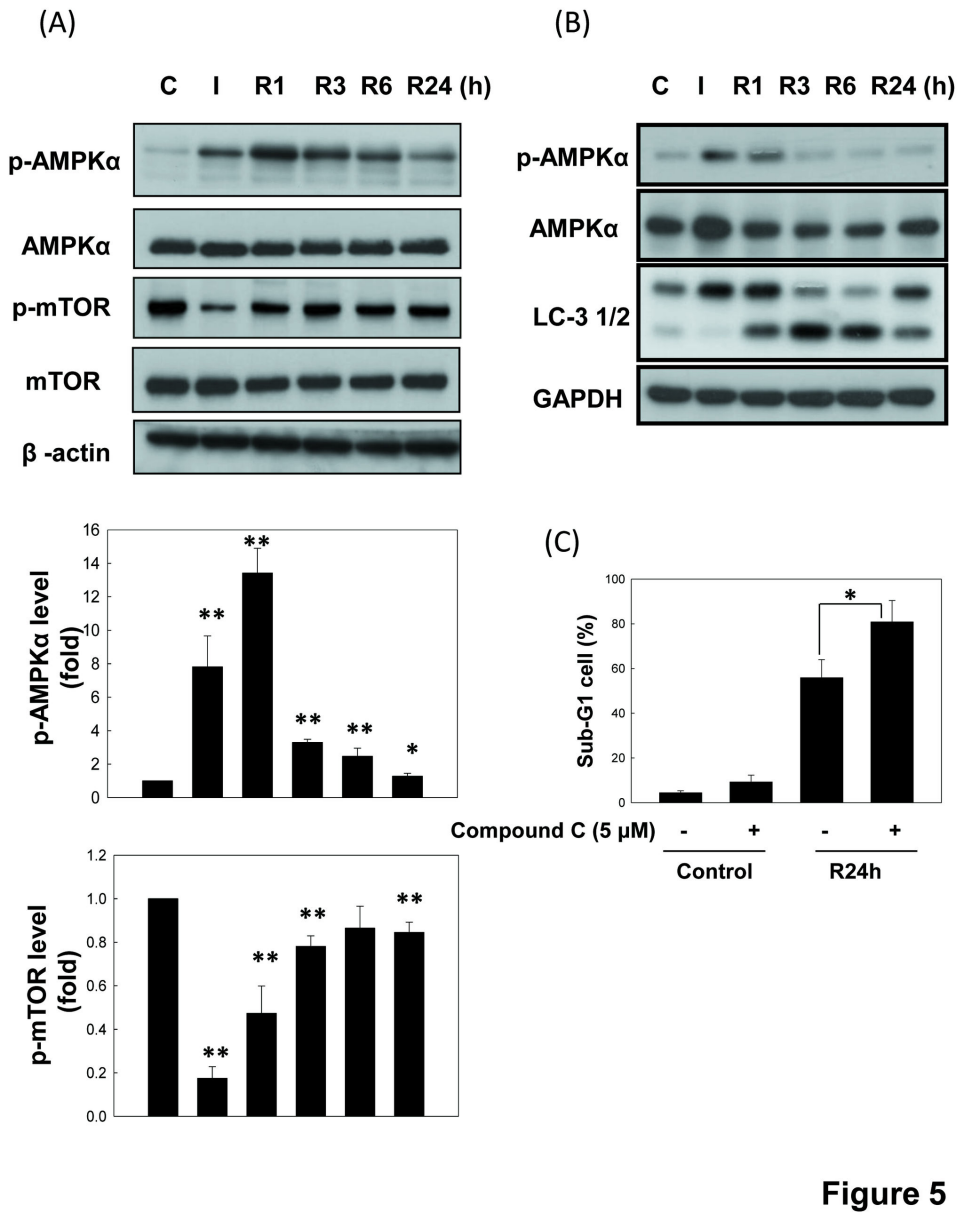
The changes in the phosphorylations of AMPK, mTOR and LC-3 cleavage in renal proximal tubule cells during *in* *vitro* I/R . LLCPK-1 (A) and NRK-52E (B and C) cells were induced ischemia (I) injury followed by reperfusion (R) for 1 to 24 h. The phospho-AMPKα, phospho-mTOR, AMPKα1, LC-3, and mTOR were determined by Western blotting. The percentages of cells, which treated with *in* *vitro* I/R and Compound C (5 μM) with the hypodiploid DNA content (sub-G1 cells) were determined by flow cytometry (C). The β-actin was used to an internal control. Data are presented as the means ± SDs in at least three independent experiments. *P < 0.05 and **P < 0.01 as compared with vehicle control group.

A previous study has reported that the energy sensor-AMPK may regulate autophagy through different downstream signals including inhibition of mTOR phosphorylation [36]. Therefore, we examined the phosphorylations of AMPK and mTOR protein during I/R. As shown in Figure 5A, the ischemia (I) caused an increase in AMPKα phosphorylation and a decrease in mTOR phosphorylation in renal cells as compared with the control cells, while the changed protein phosphorylations of AMPKα and mTOR were gradually recovered during reperfusion (R) periods. Moreover, consistent with LLC-PK1cells, NRK-52E cells presented similar tendency to AMPK phosphorylation and LC3-II changes in an *in vitro* I/R injury model (Figure 5B). Inhibition of AMPK by compound C also increased the NRK 52E cell apoptosis during I/R 24 h (Figure 5C).

**Figure 6:**
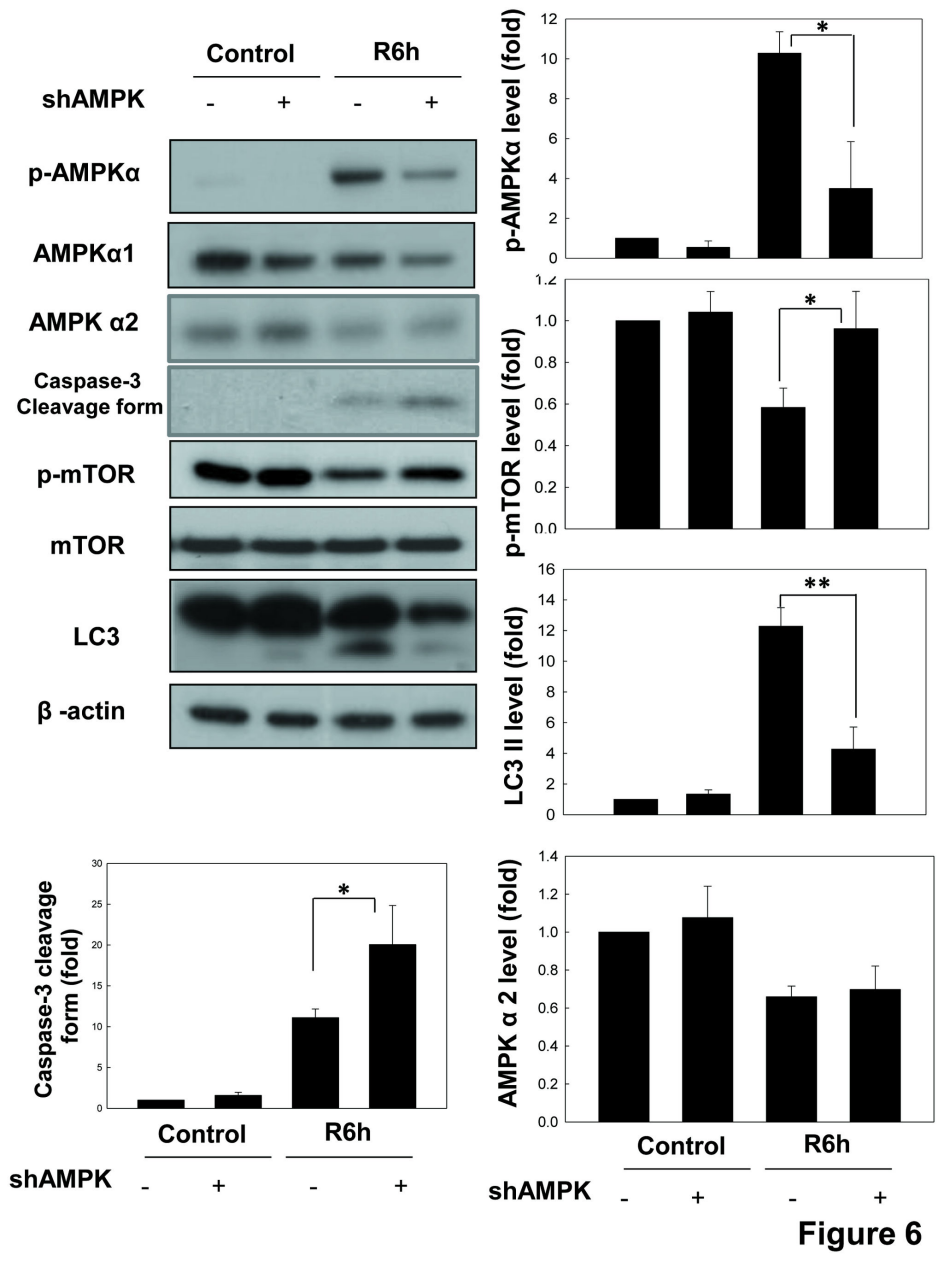
AMPK down-regulates the phosphorylation of mTOR and up-regulates the formation of LC3-II in LLC-PK1 cells during *in* *vitro* I/R. Cells, which were transfected with scrambled shRNA (scra.) or shRNA for AMPKα (shAMPK), were treated with 50 μM antimycin A and 5 mM 2-deoxyglucose for 1.5 h to induce ischemia (I) injury followed by reperfusion (R) for 6 h. The protein expressions of phospho-AMPKα, phospho-mTOR, AMPKα1, AMPKα2, mTOR, caspase-3 and LC3 were determined by Western blotting. The β-actin was used to an internal control. Data are presented as the means ± SDs in three independent experiments. *P < 0.05 and **P < 0.01 as compared with vehicle control group.

**Figure 7:**
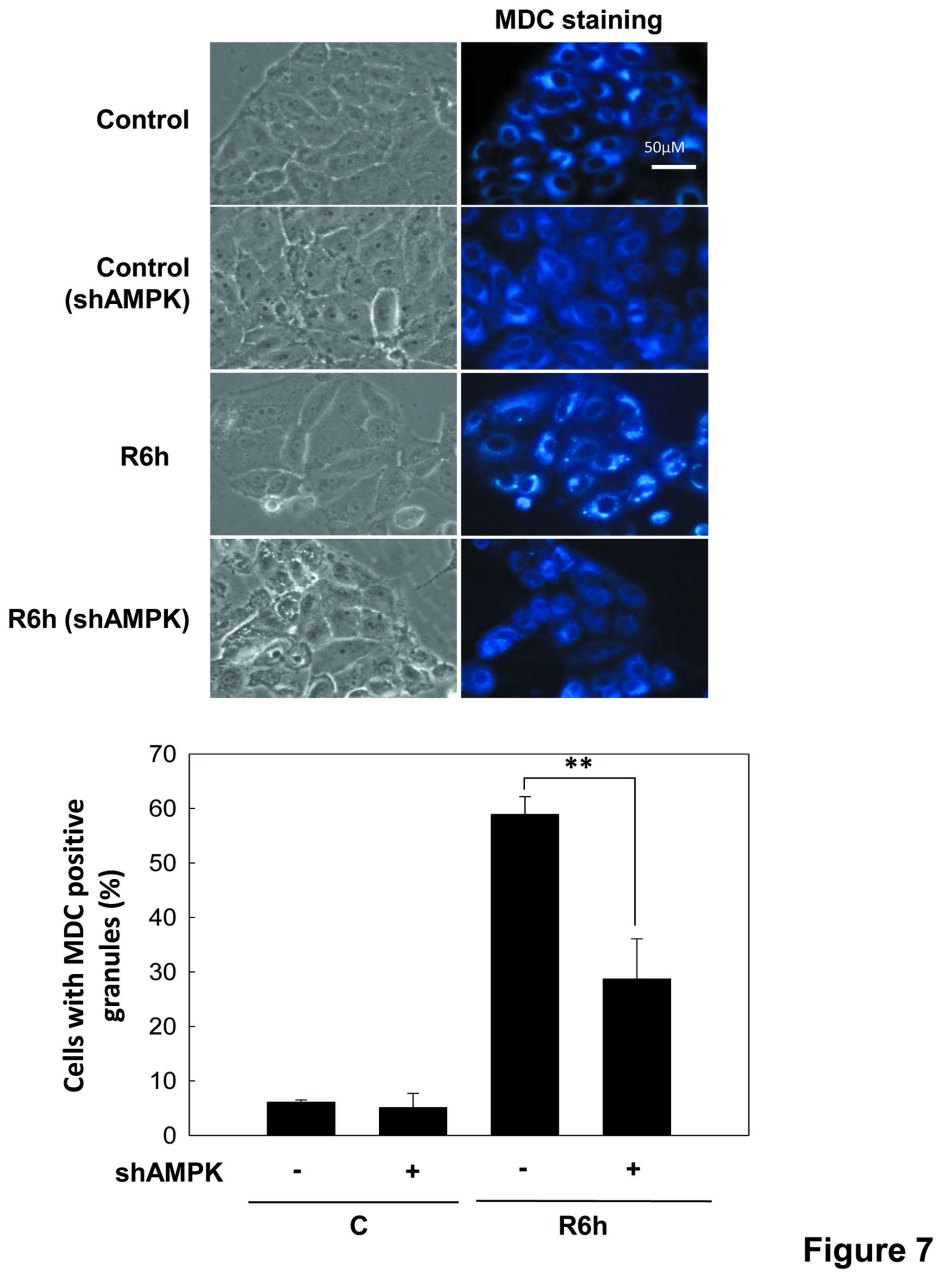
AMPK activates autophagy in LLC-PK1 cells during *in* *vitro* I/R. Cells, which were transfected with scrambled shRNA (scra.) or shRNA for AMPKα (shAMPK), were treated with 50 μM antimycin A and 5 mM 2-deoxyglucose for 1.5 h to induce ischemia (I) injury followed by reperfusion (R) for 6 h. The MDC staining for autophagic vacuoles was examined by fluorescence microscopy. Positive MDC staining cells was counted (a minimum of 100 cells per sample). Data are presented as the means ± SDs in three independent experiments. *P < 0.05 and **P < 0.01 as compared with vehicle control group.

Next, we investigated the role of AMPK and mTOR in autophagy in the renal tubular cells during *in vitro* I/R. As shown in Figure 6A, knockdown of AMPKα1 by shRNA significantly decreased the up-regulations of AMPKα phosphorylation and LC3-II formation and the down-regulation of mTOR phosphorylation in LLC-PK1 cells during I/R 6 h. There was no difference in the expression of AMPKα2 protein under AMPKα1 knockdown condition (Figure 6). Moreover, knockdown of AMPKα1 by shRNA also effectively reduced the increased MDC staining in LLC-PK1 cells during I/R 6 h. (Figure 7). Results indicated that AMPK signaling down-regulates mTOR phosphorylation and activates autophagy in the renal tubular cells during I/R injury. 

### Roles of AMPK and mTOR in apoptosis in an *in vitro* I/R injury model

**Figure 8:**
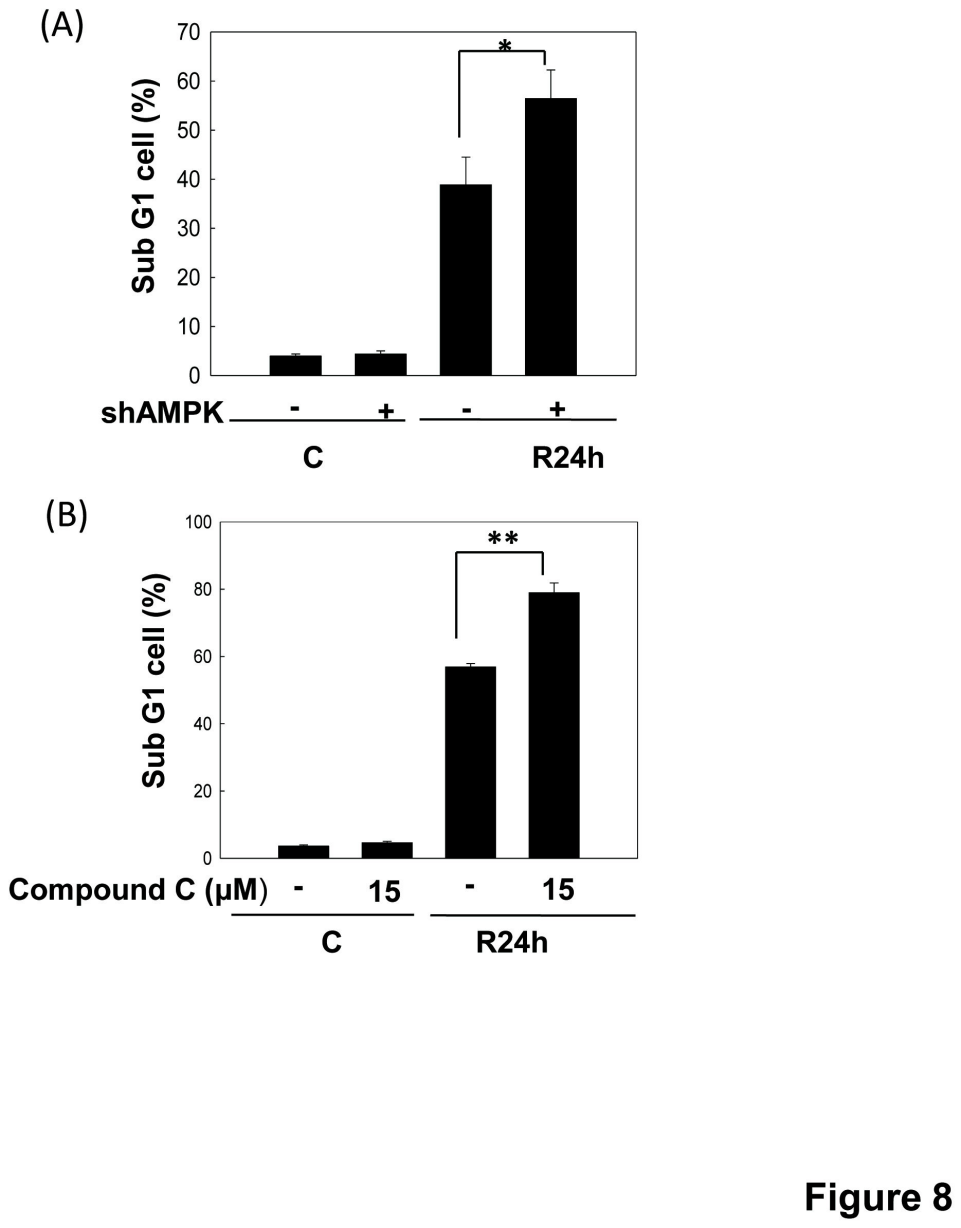
Inhibition of AMPK enhances I/R-induced apoptosis in LLC-PK1 cells. Cells, which were transfected with scrambled shRNA or shRNA for AMPKα (shAMPK) (A) or pre-treated with 15 μM Compound C (B), were treated with 50 μM antimycin A and 5 mM 2-deoxyglucose for 1.5 h to induce ischemia (I) injury followed by reperfusion (R) for 24 h. Percentages of cells with the hypodiploid DNA content (sub-G1 cells) were determined by low cytometry. Data are presented as the means ± SDs in three independent experiments. *P < 0.05 and **P < 0.01 as compared with vehicle control group.

**Figure 9:**
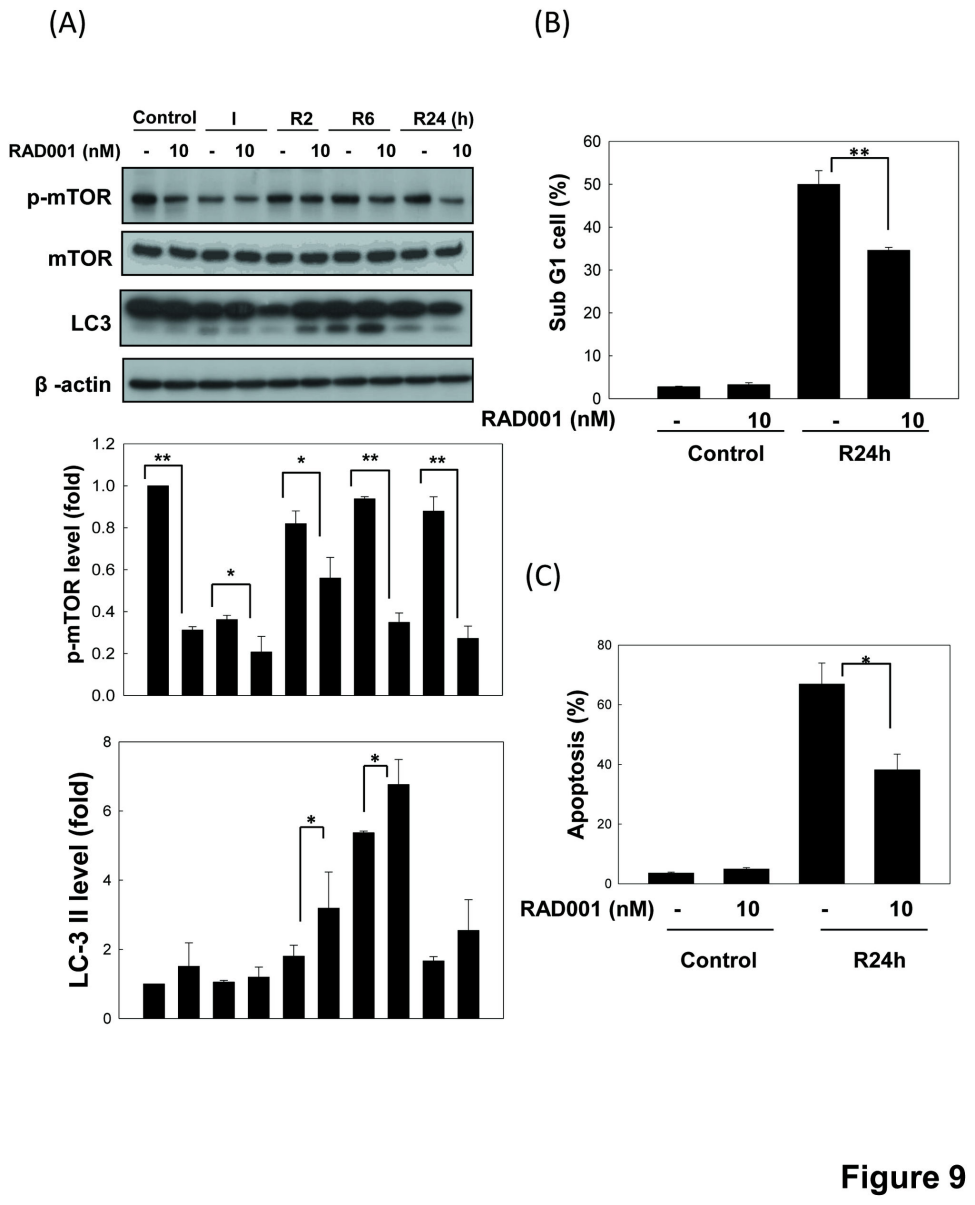
Inhibition of mTOR activates autophagy and decreases apoptosis in LLC-PK1 cells during *in* *vitro* I/R. Cells were treated with 50 μM antimycin A and 5 mM 2-deoxyglucose for 1.5 h to induce ischemia (I) injury followed by reperfusion (R) for 2-24 h in the presence or absence of 10 nM RAD001. The protein expressions of phospho-mTOR and LC3 were determined by Western blotting (A). The β-actin was used to an internal control. Moreover, percentages of sub-G1 cells and Annexin V/PI positive cells were determined by flow cytometry (B and C). All data are presented as the means ± SDs in three independent experiments. *P < 0.05 and **P < 0.01 as compared with vehicle control group.

To investigate whether AMPK protects renal cells from apoptosis during *in vitro* I/R injury, we examined the effects of AMPK inhibition by AMPKα1 shRNA and AMPK inhibitor compound C on the I/R-induced cell apoptosis. As shown in Figure 8, both AMPKα1 shRNA transfection and compound C (15 μM) treatment significantly enhanced the increased percentage of sub-G1 cells during I/R. The caspase-3 cleavage was also increased in cells with AMPK knockdown during I/R 6 h (Figure 6). It showed that AMPK activation attenuates cell apoptosis in an *in vitro* I/R injury model. Moreover, RAD001, an inhibitor of mTOR, significantly decreased mTOR phosphorylation and increased LC3-II formation in LLC-PK1 cells as compared with non-RAD001-treated cells during I/R (Figure 9A). RAD001 also effectively decreased the increased percentage of sub-G1 cells and annexin V-positive cells during I/R 24 h (Figures 9B and 9C). Results showed that AMPK-regulated mTOR signaling pathway plays a protective role in the renal tubular cell apoptosis in an *in vitro* I/R injury model. 

## Discussion

I/R is known to induce several mechanisms to cause cell repair and regeneration, which take place together with I/R-induced cellular apoptosis, autophagy, or necrosis, depending on whether cell death or regeneration prevails [37]. Recently, there is increasing evidence to suggest that autophagy participates in ischemic diseases and plays a protective role in the heart [19,36,38,39]. Consistent with previous reports, our data reveal that I/R certainly induces autophagy in renal tubular cells, and the induction of autophagy plays a protective role in the renal tubular cell injury in an *in vitro* I/R injury model. 

Autophagy has been suggested to serve as a mechanism to induce the cell survival or death, depending on the levels of cell stress [40]. The study of Xu and Zhang has indicated that cells in the brain appear to be protected under physiological levels of autophagy, which are triggered by mild to modest hypoxia/ischemia, and brain cell injury or death may be observed under high levels of autophagy induced by severe hypoxia/ischemia or I/R [41]. It has also been mentioned that autophagy plays a protective role in the development of renal injury, but excessive autophagy may cause renal cell injury or death [42]. Nevertheless, it remains unclear how autophagy protects or damages the cells during stress induction. In the present study, we demonstrated that I/R could evoke autophagy in the renal proximal renal cells in an *in vitro* I/R injury model. A pharmacological inhibitor of autophagy significantly elevated the renal tubular cell apoptosis, indicating that autophagy induction may protect renal cells from I/R injury-induced apoptosis. However, the molecular mechanisms of autophagy induction on renal I/R injury still remain unclear and need to be clarified. Moreover, the cross-regulation between autophagy and apoptosis raises a possibility that signaling activation during autophagy interferes the cell death pathway [42]. Therefore, the signaling mechanisms for autophagy induction and renal tubular cell apoptosis during I/R also need to be further explored.

Several studies have revealed that the energy sensor AMPK is capable of regulating autophagy through different downstream signalings, including mTOR, eukaryotic elongation factor-2 (eEF2) kinase, p27, and direct activation of autophagic genes [36,43]. In addition, the mTOR signaling pathway is a major positive stimulus for protein synthesis, cell growth, and cell size under a cellular stress [21]. Owing to mTOR negatively regulates autophagy, the down-regulatory effect of AMPK on mTOR signaling has been considered an important regulator of autophagy in response to energy depletion [23,43,44]. In the present study, we examined the role of AMPK and its downstream signals in I/R-induced renal tubular cell injury *in vitro*. We found that the I/R injury up-regulates the phosphorylation of AMPK and down-regulates the phosphorylation of mTOR in renal tubular cells. AMPK inhibition significantly increased the phosphorylation of mTOR as well as decreased the induction of autophagy followed by enhancing renal tubular cell apoptosis during *in vitro* I/R. Moreover, mTOR inhibition also significantly increased the enhanced autophagy and attenuated the induced renal tubular cell apoptosis during *in vitro* I/R. These findings suggest that autophagy protects the I/R-induced renal tubular cell injury via an AMPK-regulated mTOR pathway. On the other hand, the modest effect of the inhibition of autophagy by 3MA on the induction of apoptosis suggests that other factors may also be playing important roles in the I/R injury. 

LLC-PK1 is known as a renal proximal tubule cell line [45,46]. Andreucci et al. have used LLC-PK1 cells as an *in vitro* I/R model to mimic the *in vivo* renal I/R condition [47]. Results from the present study showed that the AMPK-regulated mTOR signaling pathway plays a protective role in LLC-PK1 cell apoptosis during *in vitro* I/R. To confirm the effect of I/R on LLC-PK1 cells, a rat normal renal tubular cell line NRK-52E was used. We found that AMPK phosphorylation and LC3-II formation are also increased in NRK-52E cells during *in vitro* I/R. The I/R-increased cell injury could also be enhanced by compound C. However, this chemically induced I/R cell model has its limitations and may not completely represent the *in vivo* clinical condition of I/R injury. A more direct I/R model may be needed to confirm the findings of the present study in a future study. Accordingly, we have tried to test the *in vivo* effects of I/R on renal tissues. Our preliminary data showed that AMPK phosphorylation and LC3-II formation are also increased in renal cortex of I/R-treated rats (data not shown). The detailed effects and mechanisms need to be further investigated in the future. 

## Conclusions

In this study, we demonstrated for the first time that an AMPK-regulated autophagy induction pathway plays an important protective role in the I/R-induced renal proximal tubular cell injury in an *in vitro* I/R injury model. The present study also provides the evidence that autophagy protects renal tubular cells from I/R injury through an AMPK-down-regulated mTOR signaling pathway, which may be a potential target for therapeutic intervention in the renal I/R injury.
